# The P2X7 Hypothesis of Central Post-Stroke Pain

**DOI:** 10.3390/ijms25126577

**Published:** 2024-06-14

**Authors:** Andrew Chih Wei Huang, Hsi-Chien Shih, Bai Chuang Shyu

**Affiliations:** 1 Department of Psychology, Fo Guang University, Yilan County 26247, Taiwan; 2 Institute of Biomedical Sciences, Academia Sinica, Taipei 11529, Taiwan; lahu.shi@gmail.com

**Keywords:** CPSP, P2X7 receptor, the P2X7 hypothesis of central post-stroke pain, stroke hemorrhage, mice

## Abstract

The present study examined how P2X7 receptor knockout (KO) modulates central post-stroke pain (CPSP) induced by lesions of the ventrobasal complex (VBC) of the thalamus in behaviors, molecular levels, and electrical recording tests. Following the experimental procedure, the wild-type and P2X7 receptor KO mice were injected with 10 mU/0.2 μL type IV collagenase in the VBC of the thalamus to induce an animal model of stroke-like thalamic hemorrhage. Behavioral data showed that the CPSP group induced thermal and mechanical pain. The P2X7 receptor KO group showed reduced thermal and mechanical pain responses compared to the CPSP group. Molecular assessments revealed that the CPSP group had lower expression of NeuN and KCC2 and higher expression of GFAP, IBA1, and BDNF. The P2X7 KO group showed lower expression of GFAP, IBA1, and BDNF but nonsignificant differences in KCC2 expression than the CPSP group. The expression of NKCC1, GABAa receptor, and TrkB did not differ significantly between the control, CPSP, and P2X7 receptor KO groups. Muscimol, a GABAa agonist, application increased multiunit numbers for monitoring many neurons and [Cl^−^] outflux in the cytosol in the CPSP group, while P2X7 receptor KO reduced multiunit activity and increased [Cl^−^] influx compared to the CPSP group. P2X4 receptor expression was significantly decreased in the 100 kDa but not the 50 kDa site in the P2X7 receptor KO group. Altogether, the P2X7 hypothesis of CPSP was proposed, wherein P2X7 receptor KO altered the CPSP pain responses, numbers of astrocytes and microglia, CSD amplitude of the anterior cingulate cortex and the medial dorsal thalamus, BDNF expression, [Cl^−^] influx, and P2X4 expression in 100 kDa with P2X7 receptors. The present findings have implications for the clinical treatment of CPSP symptoms.

## 1. Introduction

Central post-stroke pain (CPSP) is a kind of neuropathic pain [1]. Clinical evidence suggests that CPSP is caused by damage to the ventrobasal complex (VBC) nuclei of the thalamus (including the ventroposteriolateral and ventroposteriomedial nuclei of the thalamus), causing sensory dysfunction and spontaneous or evoked pain symptoms [2,3]. Although the prevalence rate of CPSP varies across clinical reports, it is considered to be 1–46% among patients with hemorrhagic stroke [2,3,4].

Recently, a novel view has been different from the previous pathophysiological hypotheses to explain the brain mechanisms in modulations of pain behaviors [5,6,7]. For example, the P2X7 receptor is a novel target modulated by high concentrations of adenosine triphosphate (ATP) that has been shown to control a cation channel gating Ca^2+^ and Na^+^ ions [8]. P2X7 receptors are in microglia, astrocytes, and oligodendrocytes [9]; however, whether P2X7 receptors exist in neurons is controversially discussed [8]. Extracellular ATP signaling activates chronic pain via the P2X3 receptors in primary afferent neurons and the P2X4 and P2X7 receptors activate it in spinal cord microglial cells [6]; moreover, P2X7 receptor antagonists have been developed, with therapeutic potential in neurological and psychiatric diseases [7]. P2X4 and P2X7 receptors interact with each other to reduce neuropathic pain responses [5].

On the other hand, intra-thalamus collagenase microinjections could induce persistent allodynia of mechanical pain; moreover, c-Fos, ionized calcium-binding adaptor molecule 1 (IBA1), and glial fibrillary acidic protein (GFAP) expression significantly increased in the lumbar spinal dorsal horn [10]. Chronic ischemic pain injury enhanced GFAP levels but did not significantly change IBA1 expression in the ipsilateral dorsal horn on post-injury Day 3 [11]. Chronic pain or neurological diseases reduced the levels of neuronal nuclear antigen (NeuN), but enhanced GFAP and IBA1 expressions, indicating chronic pain, and neurological diseases decreased numbers of neurons and increased numbers of astrocytes and microglia to determine the status of neuroinflammation or cell death [12,13]. Alternatively, thalamic hemorrhage-induced CPSP increased mediator complex subunit 1 (MED1) and tropomyosin-related kinase receptor B (TrkB) expression decreased brain-derived neurotrophic factor (BDNF) levels compared to the control group [14]. Lesions of the medial thalamus were associated with decreased neuron numbers and increased astrocyte numbers, microglia numbers, P2X4 receptors, and BDNF mRNA expression in the medial thalamus [15]. In electrophysiological research of neuropathic pain, spontaneous cingulate cortical high-current spikes were triggered by thalamocortical projections from the medial dorsal thalamic nuclei in chronic pain states [16]. The nociceptive inputs induced by the medial thalamus could enhance high-frequency stimulation responses in the anterior cingulate cortex (ACC) and unit activities in the medial thalamus, indicating that the medial thalamus’ projections to the ACC-mediated nociceptive stimulus-induced responses [17].

The hypothesis of chloride dysfunction-induced neuropathic pain or pathophysiological states is critical [18,19]. For example, the GABAa receptor controls chloride influx into the cytosol, resulting in hyperpolarization; oppositely, chloride outflow from the cytosol leads to neuropathic pain. The chloride gradients of the cell membrane are also controlled by KCC2 and NKCC1 active cotransporters that are correlated with the function of GABAa receptors. KCC2 is a potassium and chloride cotransporter that extrudes chloride out of neurons, while NKCC1 is a sodium, potassium, and chloride cotransporter that controls chloride influx into neurons in normal condition [19]. In the condition of neuropathic pain, NKCC1 activation induces more chloride outflow and KCC2 cotransporter increases more chloride influx [19]. According to the hypothesis of central disinhibition for neuropathic pain, the balance of chloride anions in the mechanisms of the GABAa receptor and KCC2 and NKCC1 transporters plays an important role in the occurrence of neuropathic pain and pathophysiological conditions [20]. Regardless, the involvement of the GABAa receptor, NKCC1, and KCC2 in pathophysiological states and neuropathic pain remains uncertain. For example, a previous study examined the involvement of GABAa receptors in the trigeminal processing of mechanical allodynia [21]. This study was given subcutaneous injections of interleukin-1 beta (IL-1b), and it produced mechanical and thermal pain responses. Moreover, the intracisternal administration of bicuculline, a GABAa antagonist, induced mechanical pain effects; however, intracisternal injections of bicuculline and bumetanide, an NKCC1 inhibitor, attenuated IL-1b-induced pain responses [21]. Therefore, GABAa receptors and NKCC1 were involved in IL-1b-induced pain behaviors. qRTPCR data from a chronic constriction injury model showed nonsignificant differences in KCC2 and NKCC1 expression between the chronic constriction injury and control groups, indicating that KCC2 and NKCC1 activation did not contribute to neuropathic pain [18].

The present study examined P2X7 receptor knockout (KO)-modulated pain responses (i.e., mechanical and thermal pain responses), molecular levels (i.e., NeuN, GFAP, IBA1, BDNF, TrkB, KCC2, NKCC1, and GABAa receptor), electrophysiological responses (e.g., current-source density [CSD] amplitude and peak latency, fast and late components in unit activity, and multiunit activity), and chloride ion influx into the cytosol in the MEQ staining. Moreover, it tested which site of P2X4 interacted with the P2X7 receptor in modulating CPSP. Finally, the P2X7 hypothesis of CPSP was proposed, explaining how P2X7 receptor KO modulates pain behaviors, molecular levels, electrophysiological recording, and chloride ion influx in the cytosol.

## 2. Results

### 2.1. Pain Behavioral Tests and Molecular Expression in the CPSP Group

**Figure 1:**
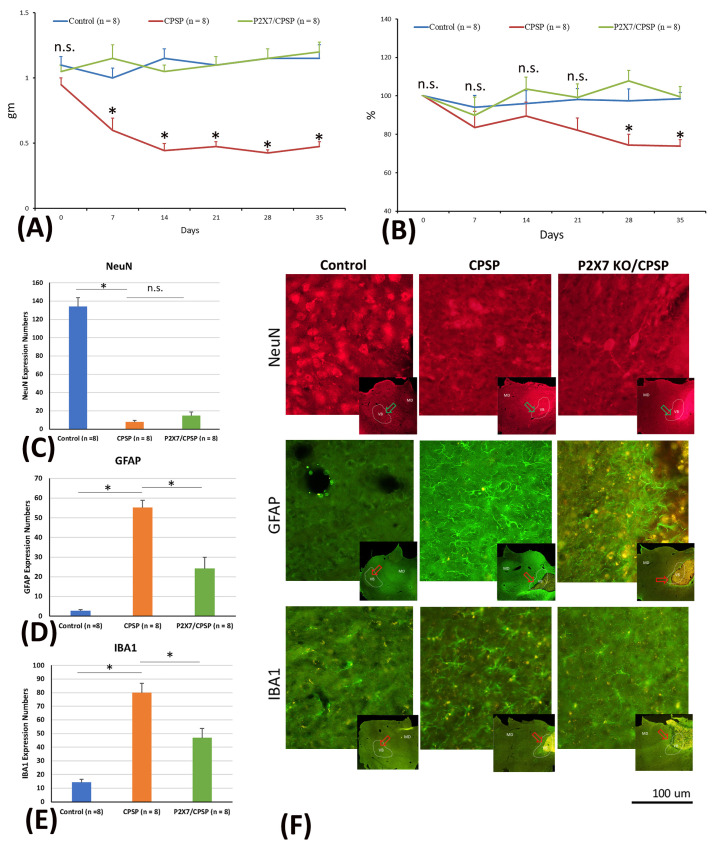
(**A**) Mechanical pain and (**B**) thermal pain were tested via the von Frey test and plantar test, respectively. Wild-type mice, wild-type mice with VBC lesions, and P2X7 KO mice with VBC lesions were respectively defined as the control, CPSP, and P2X7/CPSP groups (n = 8 per group). (**C**) NeuN expression numbers (**D**) GFAP expression numbers (**E**) IBA1 expression numbers in the medial thalamus for the control, CPSP, and P2X7/CPSP groups after behavioral tests. (**F**) In the qualitative analysis, immunofluorescence staining was performed for the expression of NeuN, GFAP, and IBA1 in the control, CPSP, and P2X7/CPSP groups. Note, gm: the threshold stimulus intensity; %: pain withdrawal latency time on test day/pain withdrawal latency time on baseline day (Day 0); Expression numbers: averaged targeting proteins numbers for each brain slice. P2X7/CPSP: P2X7 receptor knock-out mice that induced the CPSP symptoms. n.s.: nonsignificant differences. * *p* < 0.05 indicates significant differences.

To test mechanical and thermal pain responses and molecular levels, the mice were microinjected with collagenase to destroy the VBC neurons, following which mechanical pain was measured with the von Frey test and thermal pain with the plantar test. Later, immunohistochemical staining of NeuN, GAFP, and IBA1 molecules was performed. The results showed that mice with VBC lesion-induced CPSP had a decreased mechanical pain threshold and increased mechanical allodynia; however, P2X7 receptor KO mice showed an increase in this kind of pain threshold and decreased mechanical allodynia (F(2, 21) = 61.39, *p* < 0.05) on Days 7, 14, 21, 28, and 35 (F(5, 105) = 2.49, *p* < 0.05; Figure 1A).

The data of thermal pain in the plantar test showed significant differences in group (F(2, 21) = 6.54, *p* < 0.05) and group × day (F(10, 105) = 2.06, *p* < 0.05). However, nonsignificant differences occurred in day (F(5, 105) = 2.12, *p* > 0.05). The results indicated that the CPSP group showed significant decreases in the thermal pain threshold compared to the control group on Days 28 and 35 (*p* < 0.05). However, the P2X7 KO mice had a significantly increased threshold of thermal pain on all testing days compared to the CPSP group (*p* < 0.05); on the other hand, the P2X7 KO mice did not show any significant differences compared to the control group for all days (*p* > 0.05; Figure 1B).

Regarding the cell alteration tests for neuron numbers, a one-way ANOVA was conducted for NeuN expression. The results showed significant differences between groups (F(2, 21) = 133.65, *p* < 0.05). Tukey’s post hoc test indicated that the CPSP and P2X7/CPSP groups had lower NeuN expression than the control group (*p* < 0.05); however, the P2X7/CPSP group did not differ significantly from the CPSP group (*p* > 0.05). The results indicated that mice with VBC lesion-induced CPSP had decreased neuron numbers; however, P2X7 receptor KO mice did not recover the neurons after CPSP induction (Figure 1C,F upper panels).

Concerning the cell alteration tests for astrocyte numbers, a one-way ANOVA was conducted for GFAP expression. The results showed significant differences between groups (F(2, 21) = 44.22, *p* < 0.05). Tukey’s post hoc test indicated that the CPSP group had increased GFAP expression compared to the control group (*p* < 0.05); moreover, the P2X7/CPSP group’s expression was significantly decreased compared with that of the CPSP group (*p* < 0.05). The results indicated that mice with VBC lesion-induced CPSP had increased astrocyte numbers; however, the P2X7 receptor KO group had significantly decreased astrocyte numbers after CPSP induction (Figure 1D,F middle panels).

Concerning the cell alteration tests for microglia numbers, a one-way ANOVA was conducted for IBA1 expression. The results showed significant differences between groups (F(2, 21) = 32.05, *p* < 0.05). Tukey’s post hoc test indicated that the CPSP group had increased IBA1 expression compared to the control group (*p* < 0.05); moreover, the P2X7/CPSP group had significantly decreased expression compared with the CPSP group (*p* < 0.05). The results indicated that mice with VBC lesion-induced CPSP had increased microglia numbers; however, the P2X7 receptor KO group had significantly decreased microglia numbers after CPSP induction (Figure 1E,F lower panels).

### 2.2. BDNF, TrkB, KCC2, NKCC1, and GABAa Levels Measuring Pathological Status

**Figure 2:**
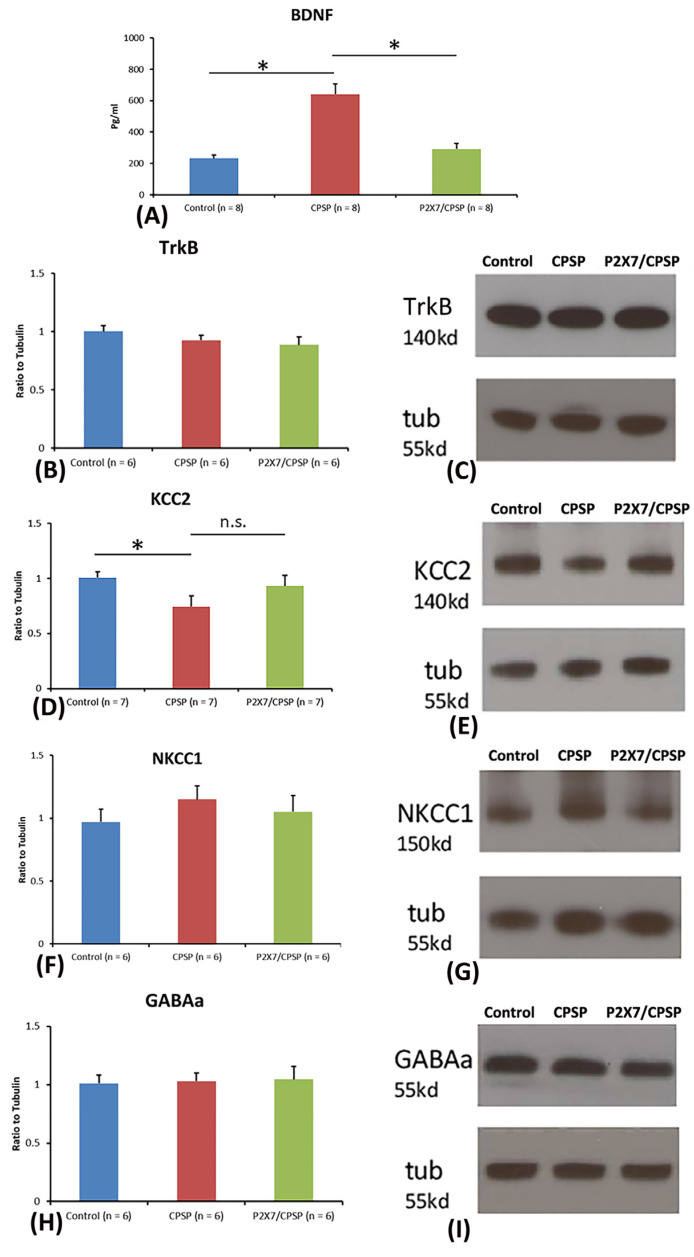
BDNF expression and channel/cotransport composition were assessed for the control, CPSP, and P2X7/CPSP groups. (**A**) BDNF expression (pg/mL) was measured for the control, CPSP, and P2X7/CPSP groups (n = 8 per group). (**B**) TrkB receptors, (**D**) KCC2 cotransport, (**F**) NKCC1 cotransport, and (**H**) GABAa receptor were measured in the medial thalamus for the control, CPSP, and P2X7/CPSP groups (n = 6–7 per group). Western blotting approaches showed specific proteins, such as (**C**) TrkB receptors, (**E**) KCC2, (**G**) NKCC1, and (**I**) GABAa receptors. P2X7/CPSP: P2X7 receptor knock-out mice that induced the CPSP symptoms. n.s.: nonsignificant differences. * *p* < 0.05 indicates significant differences.

To test BDNF and TrkB levels in the medial thalamus, a one-way ANOVA was conducted for the control, CPSP, and P2X7/CPSP groups. The factor of group had a significant effect on BDNF levels (F(2, 21) = 27.50, *p* < 0.05). Tukey’s test indicated that the BDNF levels of the CPSP group were significantly increased compared to those of the control group (*p* < 0.05); moreover, the P2X7/CPSP group had significantly lower BDNF levels compared to the CPSP group (*p* < 0.05; Figure 2A). Concerning the TrkB levels in the medial thalamus, a one-way ANOVA showed no significant differences in TrkB expression between the control, CPSP, and P2X7/CPSP groups (F(2, 15) = 1.46, *p* > 0.05; Figure 2B,C).

Regarding KCC2, NKCC1, and GABAa expression, a one-way ANOVA was performed for the control, CPSP, and P2X7/CPSP groups. The results showed significant differences between groups (F(2, 18) = 3.97, *p* < 0.05). Tukey’s test indicated that the CPSP group had a significant decrease in KCC2 expression compared to the control group (*p* < 0.05); however, no significant differences between the CPSP and P2X7/CPSP groups were observed (*p* > 0.05; Figure 2D,E). Concerning NKCC1 and GABAa expression, a one-way ANOVA indicated no significant differences in NKCC1 (F(2, 15) = 0.76, *p* > 0.05; Figure 2F,G) and GABAa (F(2, 15) = 0.05, *p* > 0.05; Figure 2H,I) levels between the control, CPSP, and P2X7/CPSP groups.

### 2.3. Assessments of Nociceptive Neuronal Responses in the Anterior Cingulate Cortex (ACC) and Medial Dorsal Thalamus (MD) of CPSP and P2X7 Receptor KO Mice

**Figure 3:**
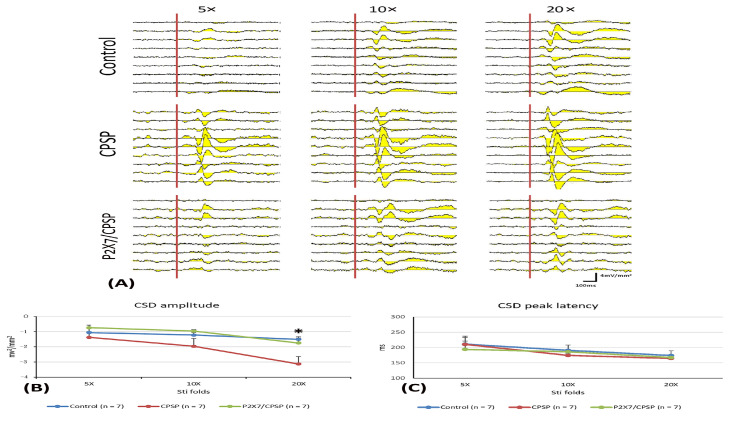
(**A**) ACC electrophysiological recording with 5-, 10-, and 20-fold CSD amplitude to nociceptive responses in the control, CPSP, and P2X7/CPSP groups (n = 7 per group). (**B**) Serials of CSD amplitude (mv^2^/mm^2^) and (**C**) CSD peak latency (ms) in ACC with 5-, 10-, and 20-fold basic stimulation strength electric stimulation on the sciatic nerve for the control, CPSP, and P2X7/CPSP groups. ACC: anterior cingulate cortex; CSD: current source density. P2X7/CPSP: P2X7 receptor knock-out mice that induced the CPSP symptoms. * *p* < 0.05 indicates significant differences.

**Figure 4:**
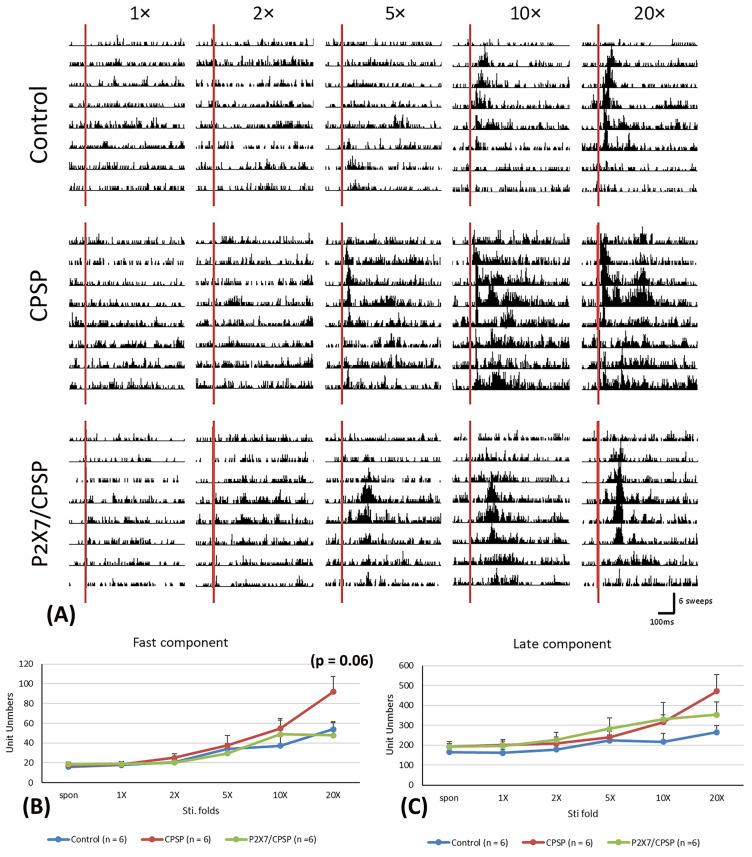
(**A**) MD electrophysiological recording with 1-, 2-, 5-, 10-, and 20-fold nociceptive responses after repeated noxious electric stimuli on the sciatic nerve in the control, CPSP, and P2X7/CPSP groups (n = 6 per group). Electrophysiological data of multiunit numbers in MD with 1- to 20-fold basic electric stimulations on the sciatic nerve in the control, CPSP, and P2X7/CPSP groups for (**B**) fast component and (**C**) late component. MD: mediodorsal nucleus of the thalamus. P2X7/CPSP: P2X7 receptor knock-out mice that induced the CPSP symptoms.

MD and ACC were main central function areas of the pain/noxious pathway in humans and rodents. To evaluate noxious responses in normal and chronic pain states, multi-channel electrodes were inserted into the MD and ACC. An electric shock stimulator implanted on the contra lateral sciatic nerve. The noxious response was induced by electric shock on the sciatic nerve (Figure 3 and Figure 4, see details in Methods).

To elucidate the CSD amplitude and CSD peak latency in the ACC, 5×, 10×, and 20× stimulation folds were tested (Figure 3A). The results indicated that the 20× stimulation fold condition was significantly increased for the CPSP group compared to the control group (F(2, 18) = 6.28, *p* < 0.05, following one-way ANOVA analysis); however, the other stimulation folds were not significantly different between the control, CPSP, and P2X7/CPSP groups (*p* > 0.05, following the post hoc tests with Tukey; Figure 3B).

Regarding the CSC latency test, the results showed nonsignificant differences in group (F(2, 18) = 0.17, *p* > 0.05) and group × stimulation fold (F(4, 36) = 0.63, *p* > 0.05); significant differences occurred in stimulation fold (F(2, 36) = 12.11, *p* > 0.05) for the CDS peak latency (Figure 3C).

To examine the electrical recording for the fast component in the MD, 1×, 2×, 5×, 10×, and 20× stimulation folds were tested (Figure 4A). The results indicated that significant differences occurred in stimulation fold (F(5, 75) = 23.70, *p* < 0.05) and group × stimulation fold (F(10, 75) = 2.00, *p* < 0.05); however, there was no significant difference in group (F(2, 15) = 1.60, *p* > 0.05) in the fast component test. The unit numbers of fast component analysis with Tukey’s post hoc test indicated that the 20× stimulation fold condition was likely increased for the CPSP group compared to the control group (*p* = 0.06); however, the P2X7/CPSP group did not differ significantly from the CPSP group (*p* > 0.05; Figure 4B).

Regarding the late component test in the MD, the results showed nonsignificant differences in group (F(2, 15) = 1.55, *p* > 0.05) and group × stimulation fold (F(10, 75) = 1.57, *p* > 0.05) and significant differences in stimulation fold (F(5, 75) = 14.63, *p* < 0.05; Figure 4C).

### 2.4. Thalamic GABAergic Inhibition Remained in P2X7 KO Mice after Thalamic Lesion

**Figure 5:**
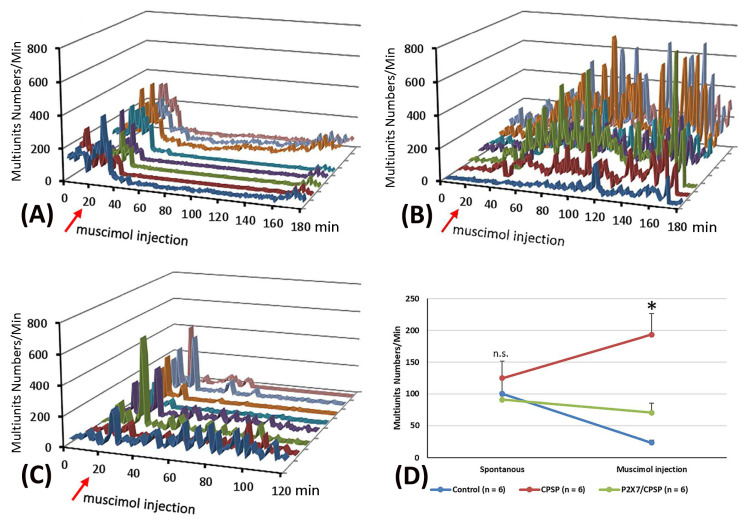
Effect of muscimol, a GABA agonist, on the control, CPSP, and P2X7/CPSP groups. Multiunit numbers per minute were measured after muscimol application for the (**A**) control, (**B**) CPSP, and (**C**) P2X7/CPSP groups over 180 min. (**D**) Multiunit numbers per minute were assessed for the control, CPSP, and P2X7/CPSP groups (n = 6 per group) during the spontaneous and muscimol application phases. P2X7/CPSP: P2X7 receptor knock-out mice that induced the CPSP symptoms. Note, different color of lines indicates different electrical channels. n.s.: nonsignificant differences. * *p* < 0.05 indicates significant differences.

Concerning the pattern analysis of the multiunit numbers, the control group showed that the silenced state was permanent for more than 60 min (Figure 5A). In the CPSP group, the multiunit numbers of the thalamus presented a chronic excitation over 180 min after muscimol injection (Figure 5B). Compared to the CPSP group, P2X7 KO mice with a VBC lesion did not show the abnormal excitation induced by muscimol microinjection (Figure 5C).

To evaluate the function of GABA inhibition in the thalamus, muscimol, an agonist of the GABAa receptor, was microinjected into the thalamus. Moreover, the pre- and post-microinjection statuses were assessed regarding the multiunit numbers for MD neuronal activity. The results showed that significant differences occurred in group (F(2, 15) = 7.95, *p* < 0.05) and group × drug injection (F(2, 15) = 12.49, *p* < 0.05); however, nonsignificant differences occurred in drug injection (F(1, 15) = 0.66, *p* > 0.05). Tukey’s test indicated that the multiunit numbers of the CPSP group significantly increased compared to the control group (*p* < 0.05); moreover, the P2X7/CPSP group showed a significant decrease in multiunit numbers compared to the CPSP group (*p* < 0.05) with muscimol microinjection treatment (Figure 5D).

### 2.5. Examination of the Flow of Cytoplasmic Chloride Evoked by Muscimol Injection

**Figure 6:**
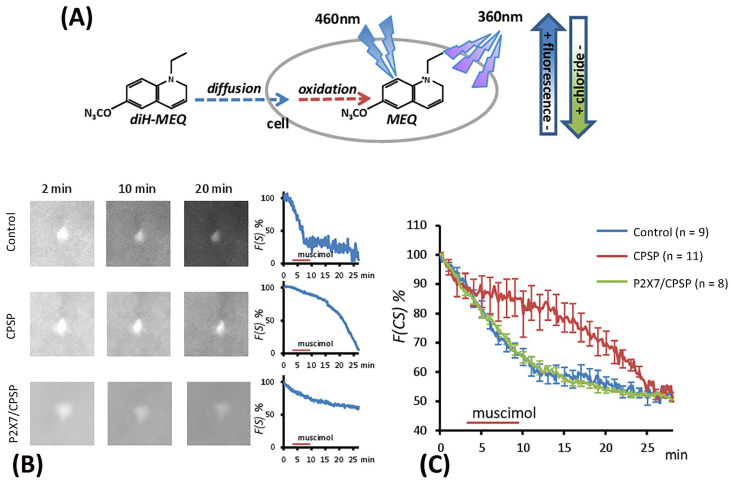
The flow of cytoplasmic chloride (F(CS)%) evoked by muscimol application was assessed for the control (n = 9), CPSP (n = 11), and P2X7/CPSP (n = 8) groups. (**A**) The process of MEQ infusion into the brain slice and the rationale for assessing chloride amounts. (**B**) Example images of MEQ fluorescence expression of the control, CPSP, and P2X7/CPSP groups. The strength of MEQ fluorescence gradually declined by light shooting. The chloride ion channel was opened by muscimol induction, and the immediate [Cl^−^]^in^ change was shown by MEQ fluorescence measurement. (**C**) The F(CS)% of the control, CPSP, and P2X7/CPSP groups was assessed after muscimol application over 25 min. P2X7/CPSP: P2X7 receptor knock-out mice that induced the CPSP symptoms.

The excitation induced by muscimol was assumed to be due to abnormal [Cl^−^]^in^ accumulation resulting from the deficit of KCC2 cotransport expression following VBC lesion-induced CPSP. The present study employed the MEQ staining method to monitor the [Cl^−^]^in^ outflow (Figure 6A). The recovered brain slice was immersed in MEQ/aCSF solution for 2, 10, and 20 min to facilitate cell absorption of MEQ in the control, CPSP, and P2X7/CPSP groups (Figure 6B). To measure the [Cl^−^]^in^ outflow mediated by the GABAa channel, the incubation of the aCSF solution was perfused with muscimol/aCSF (100 µM/4 mL/min) for 5 min and then washed with aCSF solution. After the serial light excitation, the reflection strength of the light was gradually decreased due to the decay of [MEQ]^in^ (Figure 6B).

To test the [Cl^−^]^in^ outflow induced by the GABAa channel, a 3 × 30 mixed two-way ANOVA was conducted for the control, CPSP, and P2X7/CPSP groups. The results showed significant differences in group (F(2, 25) = 5.07, *p* < 0.05), time (F(29, 25) = 117.17, *p* < 0.05), and group × time (F(58, 725) = 4.48, *p* < 0.05) in the relative intensity to MEQ. Tukey’s post hoc test indicated that the CPSP group had a significant decrease in [Cl^−^]^in^ outflow compared to the control group (*p* < 0.05); however, the P2X7/CPSP group had a significant increase in [Cl^−^]^in^ outflow compared to the CPSP group (*p* < 0.05) over 10–20 min (Figure 6C).

Therefore, the present data showed that abnormal [Cl^−^]^in^ accumulation did not occur in the P2X7/CPSP group, indicating that [Cl^−^]^in^ was still in a normal state in P2X7 KO mice with a VBC lesion. The previous data indicated that the [Cl^−^]^in/out^ balance was determined by the expression of KCC2, which may also influence the function of the GABA system [19].

### 2.6. P2X4 Expression Assessments in CPSP and P2X7 Receptors

**Figure 7:**
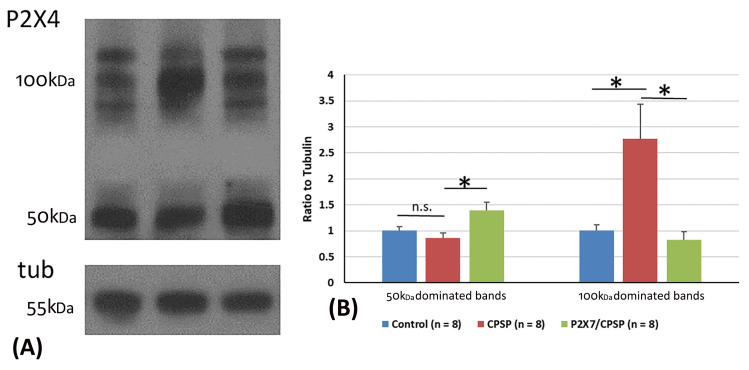
Overexpression of the P2X4 receptor after VBC lesions did not occur in P2X7 KO mice. (**A**) Examples of Western blotting results for P2X4 of the control, CPSP, and P2X7/CPSP groups. Note that monomeric P2X4 is distributed at 50 kDa and its dimeric form is at ~100 kDa. (**B**) Ratio to tubulin for 50 kDa and 100 kDa dominated bands for the P2X4 receptor in the control, CPSP, and P2X7/CPSP groups (n = 8 per group). n.s.: nonsignificant difference; *: significant difference. P2X7/CPSP: P2X7 receptor knock-out mice that induced the CPSP symptoms.

P2X4 overexpression was crucial for VBC lesion-induced CPSP symptoms. Considering the previous data, two major P2X4 bands, including 50 kDa and 100 kDa dominating bands, were observed via Western blotting (Figure 7A).

For the analysis of P2X4 expression in 50 kDa bands, one-way ANOVA was conducted for the control, CPSP, and P2X7/CPSP groups. The factor of group showed significant differences in the ratio to tubulin (F(2, 21) = 5.76, *p* < 0.05). Tukey’s test indicated that the CPSP group was not significantly different compared to the control group (*p* > 0.05). However, the P2X7/CPSP group had a significant increase in the ratio to tubulin compared to the CPSP group (*p* < 0.05; Figure 7B).

For the analysis of P2X4 expression in the 100 kDa bands, a one-way ANOVA was conducted for the control, CPSP, and P2X7/CPSP groups. The factor of group showed significant differences in the ratio to tubulin (F(2, 21) = 7.16, *p* < 0.05). Tukey’s post hoc test indicated that the CPSP group had a significant increase compared to the control group (*p* < 0.05). Moreover, the P2X7/CPSP group had a significant decrease in the ratio to tubulin compared to the CPSP group (*p* < 0.05; Figure 7B).

## 3. Discussion

Previous evidence shows that few studies examined whether P2X7 receptors are involved in the pain symptoms of CPSP. The present study is the first to test the hypothesis that P2X7 receptors contribute to CPSP mechanisms in terms of behaviors, molecular levels, electrophysiological recordings, chloride ion levels inside neurons, and interaction with P2X4 receptors: the hypothesis was named the P2X7 hypothesis of CPSP (see Table 1).

### 3.1. The P2X7 Receptor and Pain Responses

The present results showed that CPSP mice had a decreased mechanical pain threshold and hypersensitivity to mechanical pain, similar to pain symptoms in human studies [2,3,4]. These data from the CPSP animal model were consistent with previous evidence [22,23]. For example, animals with thalamic hemorrhage mimicking human stroke hemorrhage could produce thermal and mechanical pain responses [23]. VBC lesions induced pain responses in the acquisition and retrieval phases. Moreover, VBC lesion-induced CPSP did not affect motor function and explicit memory in spatial learning; however, it facilitated morphine-induced conditioned place preference learning in implicit memory [22]. The NLRP3 inflammasome was found to promote CPSP pain responses [24]. Tricyclic antidepressants and specific serotonin reuptake inhibitors have been demonstrated to decrease CPSP pain responses [25].

**Table 1:** The P2X7 hypothesis of CPSP summarizing the results of the CPSP and P2X7 receptor KO groups regarding mechanical and thermal pain, molecular assessments, electrophysiological recordings in the ACC and MD, and P2X4 receptor labeling.

	Previous CPSP Data	Previous P2X7/CPSP Data	CPSP	P2X7/CPSP
Pain behavior				
Von Frey test (mechanical)	↑ [2,3,4]	↓ [5]	↑	↓
Plantar test (thermal)	↑ [23]	↓ [5]	↑	↓
Molecular assessments				
NeuN (neuron)	↓ [10]	−−	↓	−−
GFAP (astrocyte)	↑ [10,11]	−−	↑	↓
IBA1 (microglia)	↑ [10]	−−	↑	↓
BDNF	i.c. [14,15]	−−	↑	↓
TrkB	↑ [14]	−−	n.s.	n.s.
KCC2	↓ [15]	−−	↓	n.s.
NKCC1	i.c. [15,18,21]	−−	n.s.	n.s.
GABAa	i.c. [15,18,21]	−−	n.s.	n.s.
EEG: ACC recording				
CSD amplitude	↑ [26]	↓ [26]	↓	↑
CSD peak latency	−−	−−	n.s.	n.s.
EEG: MD recording for unit numbers				
Fast component	↑ [16,17]	↓ [16,17]	↑	↓
Late component	−−	−−	n.s.	n.s.
Muscimol applications				
Multiunit	−−	−−	↑	↓
F(CS)%: [Cl^−^] inside neurons	−−	−−	↑	↓
P2X4 expression		−−		
50 kb	−−	−−	n.s.	↑
100 kb	−−	−−	↑	↓

Note: CPSP: central post-stroke pain; ACC: anterior cingulate cortex; MD: medial dorsal nucleus of the thalamus; n.s.: nonsignificant difference; ↑: significant increase; ↓: significant decrease; the CPSP group was compared to the control group; the P2X7/CPSP group was compared to the CPSP group.

However, the present study showed that P2X7 receptor KO mice with CPSP recovered a mechanical pain threshold like that of the control group in the von Frey test. The CPSP mice seemingly had a decreased thermal pain threshold; moreover, P2X7 receptor KO mice with CPSP had an increased thermal pain threshold in the plantar test, indicating that P2X7 KO mice with CPSP had decreased thermal and mechanical hypersensitivities in pain responses. These findings support previous data [5]. For example, neuropathic pain was reduced by a P2X4 and P2X7 receptor antagonist [5,26]. P2X7 receptor activation facilitated CPSP-induced pain responses and was associated with increases in neuroinflammatory cytokine levels; moreover, the administration of A740003, a P2X7 antagonist, disrupted pain responses and neuronal activity in the spinothalamocortical pathway [26]. Furthermore, P2X7 receptor antagonism is a potential treatment strategy for neuropathic pain, neurological diseases, and psychiatric disorders [7]. Therefore, P2X7 is involved in pain responses induced by thalamic hemorrhage; moreover, P2X7 receptor antagonists might be alternative treatments for the amelioration of neuropathic pain.

### 3.2. P2X7 Receptors: Neurons, Astrocytes, and Microglia

The present data show lower NeuN expression for neuron numbers, higher GFAP expression for astrocyte number, and higher IBA1 expression for microglia number in the CPSP group, seemingly to induce neuroinflammation responses in neurons and glial cells after VBC lesion-induced CPSP symptoms. Interestingly, the P2X7 receptor KO group ameliorated the neuroinflammation: P2X7 receptor KO reduced GFAP and IBA1 levels in the group of P2X7 receptor KO mice with CPSP when compared to the CPSP group. The present data of the CPSP group were consistent with previous evidence [10,11]. For example, microinjections of collagenase into the thalamus produced chronic allodynia of mechanical pain, and significantly increased c-Fos, IBA1, and GFAP expression in the lumbar spinal dorsal horn [10]. Chronic ischemia-induced pain facilitated GFAP levels but led to nonsignificant differences in IBA1 expression in the ipsilateral dorsal horn on post-injury days [11]. Therefore, VBC lesion-induced CPSP could promote neuroinflammatory responses in neurons and glia; moreover, P2X7 receptor KO could alleviate these responses.

### 3.3. P2X7 Receptors: BDNF and TrkB

BDNF is a neurotrophin derived and secreted from neurons [27]. The functions of BDNF are related to neuronal survival, neuronal growth, and synaptic plasticity [28]. BDNF plays a potential role in pathological diseases [29] and the treatment of psychiatric disorders [28]. BDNF involvement in CPSP symptoms remains uncertain. For example, an animal study of thalamic hemorrhage showed that hippocampal TrkB was significantly increased but BDNF was significantly decreased in the CPSP group [14]. However, another study revealed that BDNF expression was significantly increased in the medial thalamus of the CPSP group, while GABAa receptor and KCC2 cotransporter had lower expression in the medial thalamus of the CPSP group [15]. The present study showed that BDNF expression was increased in the medial thalamus for the CPSP group; however, some research revealed the inconsistent results that BDNF had lower expression in the CPSP group [14]. The discrepancy data might result from testing the different brain areas, such as BDNF decreases in the hippocampus [14] and increases in the medial thalamus [15]. Therefore, these data meant that the CPSP animals seemingly reduced the functions of the hippocampus and increased hyperactivity in the medial thalamus. These discrepancy findings for BDNF expression need to be scrutinized further.

On the other hand, no research examined how P2X7 receptor antagonism affects CPSP symptoms and the association with BDNF levels. Accordingly, our study was the first to examine whether P2X7 receptor KO affects CPSP symptoms and BDNF levels.

In summary, the inconsistencies in the findings regarding whether CPSP animals showed higher or lower BDNF levels should be examined further. Alternatively, the role of P2X7 receptor-mediated BDNF-TrkB signaling in CPSP symptoms should be examined further.

### 3.4. P2X7 Receptors: KCC2, NKCC1, and GABAa Receptors

According to previous evidence on CPSP, the disinhibition hypothesis suggested that individuals experienced neuropathic pain resulting from the disinhibition of the medial thalamus from the spinal thalamic tract in the spinothalamocortical pathway [2]. This kind of disinhibition of the medial thalamus is governed by the imbalance of [Cl^−^] influx and [Cl^−^]outflux in the cytosol. [Cl^−^] outflux overload outside the cytosol caused depolarization, resulting in neuropathic pain in CPSP patients. Two cotransporters, KCC2 and NKCC1, and the GABAa receptor were involved in the cellular mechanism of the disinhibition hypothesis of CPSP in that the chloride gradients were controlled by KCC2 and NKCC1 [19]. In the development and normal condition, the GABAa receptor governs chloride ion influx into the cytosol, whereas KCC2 is a potassium and chloride cotransporter that extrudes chloride out of neurons [19]. In normal conditions, NKCC1 is a sodium, potassium, and chloride cotransporter that controls more chloride influx into neurons [19]. As the NKCC1 cotransporter is activated to increase more chloride ion outflow, GABAa receptors exclude chloride ions outside the cytosol [19]. Therefore, the cell membrane induced depolarization, the so-called pathological condition. This pathological condition may be one of the reasons for CPSP pain symptoms.

Concerning whether NKCC1, KCC2, and GABAa receptors are involved in CPSP symptoms, previous data were inconsistent [15,18,21]. For example, subcutaneous injections of IL-1b could induce mechanical and thermal pain responses. Bicuculline, a GABAa antagonist, also induced mechanical pain responses following intracisternal administration; however, intracisternal injections of bicuculline and bumetanide, a NKCC1 inhibitor, interfered with IL-1b-induced pain responses [21]. The results indicated that GABAa receptors and the NKCC1 cotransporter were involved in IL-1b-induced pain responses. In our recent study, BDNF expression was significantly increased in the medial thalamus of the CPSP group, but KCC2 cotransporters were significantly decreased in the medial thalamus of the CPSP group, indicating that BDNF, the GABAa receptor, and KCC2 contributed to CPSP symptoms [15]. Nevertheless, another study demonstrated nonsignificant differences in KCC2 and NKCC1 expression between the chronic constriction injury and control groups in qRTPCR tests, indicating that KCC2 and NKCC1 were not involved in the neuropathic pain animal model of chronic constriction injury [18]. In the present study, NKCC1 and GABAa receptors were not involved in the CPSP and P2X7/CPSP groups; however, KCC2 levels were lower in the CPSP group, although no significant differences were observed between the CPSP and P2X7/CPSP groups. Obviously, the present data did not support the previous evidence. This discrepancy between our KCC2 result and the previous data is probably due to the insufficient numbers of observations in our experiment. This issue needs to be investigated further.

In summary, the issue of whether NKCC1, KCC2, and GABAa modulate CPSP and P2X7 receptor KO with CPSP should be examined in further studies.

### 3.5. P2X7 Receptors: ACC and Medial Dorsal Thalamus CSD Amplitude and CSD Peak Latency in CPSP Electrophysiological Recordings

Previous studies demonstrated that the nociceptive inputs induced by the medial thalamus (MT) could enhance high-frequency stimulation responses in the ACC and unit activities in the medial thalamus, indicating that the medial thalamus projections to the ACC mediated nociceptive stimulus-induced responses [16,17]. A recent study suggested that evoked higher CSD responses in the ACC and unit activity in the MT occurred for the CPSP group; however, a P2X7 antagonist was applied in the MT, and the ACC’s CSD and the MT unit activity were significantly decreased [26].

However, the present data were not consistent with these previous findings. For example, the present electrophysiological recordings in the ACC indicated that the CPSP group decreased CSD amplitude; moreover, P2X7 KO mice recovered the reduction of the CSD amplitude, especially for the 20-fold stimulus. The data indicated that VBC lesions in the thalamus induced CSD amplitude decreases, but P2X7 receptor KO increased CSD amplitude in the ACC compared to the CPSP group. Alternatively, the MD recordings showed that the fast component was likely increased for the CPSP group, and the P2X7/CPSP group decreased the unit numbers of the fast component. Therefore, why did the MD unit activity oppose the CSD in the ACC in our data and show congruence with the previous evidence? This should be scrutinized in further studies.

In addition, application of the GABAa agonist muscimol in the MD enhanced multiunit activity in the thalamus of the CPSP group; however, P2X7 receptor KO mice with muscimol application decreased the enhancement seen in the CPSP group in multiunit numbers in the thalamus and rescued the multiunit numbers to those of the control group. The results indicated that the GABAa agonist muscimol, affiliated with GABAa receptors, opened chloride ion channels, and promoted chloride ion influx into the neuronal membrane. The chloride influx induced hyperpolarization and led to CPSP symptoms; however, P2X7 receptor KO decreased multiunit numbers in the thalamus, resulting in reduced pain responses.

### 3.6. Chloride Influx in the Cytosol: Pain Behaviors and P2X7 Receptors

The data from the MEQ staining method showed that muscimol application decreased chloride ion inflow in the CPSP group; however, chloride ion inflow was increased in the P2X7/CPSP group compared to the CPSP group. The chloride ion inflow was not significantly different for the P2X7 KO mice with the VBC lesion compared to the control group. Therefore, the MEQ staining data supported the disinhibition hypothesis suggesting that higher chloride outflow in the cytosol induced depolarization causing CPSP pain symptoms; however, P2X7 receptor KO decreased the higher concentration of the chloride ion outflow, resulting in reduced pain responses.

### 3.7. P2X7 and P2X4 Receptors in Pain

A growing body of evidence has shown that P2X4 and P2X7 receptors [6,30,31] and, moreover, interactions between P2X4 and P2X7 receptors [32] contribute to neuropathic pain via the spinal microglia. However, no research examined the mechanism of interaction. The present study addressed this issue and found via analysis of P2X4 expression in 50 kb and 100 kb bands that the CPSP group had no significant differences in P2X4 expression in 50 kb compared to the control; however, the P2X7/CPSP group had increased P2X4 expression in 50 kb. On the other hand, the CPSP group showed a significant increase in P2X4 expression in 100 kb; moreover, P2X7 receptor KO mice recovered P2X4 expression in 100 kb. The data indicated that P2X7 interacted with P2X4 receptors in 100 kb. CPSP induced the overexpression of P2X4 receptors; however, P2X7 receptor KO decreased P2X4 receptor expression, especially in 100 kb. Therefore, the 100 kb band of P2X4 receptors was a seemingly critical site of interaction with P2X7 receptors for modulating the pain responses of CPSP.

### 3.8. Experimental Limitations and Further Studies

The present data of P2X4 receptors using the Western blot approach showed the results of double bands. Why did P2X4 induce the crosslinking, resulting in the double bands? Some research showed similar results. For example, a cell culture study demonstrated that applying disuccinimidyl suberate (DSS), a cross-linker, to the microglia produced a double band between 100–160 KDa for P2X7 receptor proteins [33]. The study with the DSS method indicated that the preferred assembly pathway for both receptors was the formation of homotrimers; moreover, homotrimers of P2X7 could co-immunoprecipitate with P2X4, and thereby an interaction occurred for P2X7 and P2X4 between, rather than within, receptor complexes [33]. Thus, this study used DSS to induce the crosslinking effect for P2X4. In our study, it may be due to the reason that CPSP-induced continuous stress and pain which triggered the double band effect. This issue of why P2X4 revealed the crosslinking and double band effects should be examined for further studies.

On the other hand, the present study examined the CPSP effect and P2X7 receptor KO effect in various assessments. However, it did not have the P2X7 KO/control group. If the experimental design was the control, CPSP, P2X7 KO/control, and P2X7 KO/CPSP four groups, the results would show the main effect of CPSP, the main effect of P2X7 KO, and the interaction of CPSP and P2X7 KO. However, we did not want to test the interaction of CPSP and P2X7 KO. We only want to test the effect of CPSP and the effect of P2X7 KO. Therefore, the present study was designed to test the control, CPSP, and P2X7 KO/CPSP three groups.

An emerging issue of concern should be whether P2X7 KO/control induced some effects that affected the results of the present study after adding the P2X7 KO/control group. Based on previous studies, P2X7 antagonism or P2X7 KO decreased the severity of inflammatory responses, and it can serve as a potential novel anti-inflammatory therapy [34]. P2X7 receptors were involved in inflammation, immune cell responses, and the gastrointestinal system under physiological and pathophysiological conditions [35]. Moreover, P2X7 receptors modulated inflammation and neuropathic pain states [36]. Therefore, the P2X7 KO/control group might modulate neuropathic pain and inflammatory responses. The lack of a P2X7 KO/group is likely to be an experimental limitation, and it should be considered for adding to further studies.

### 3.9. The P2X7 Hypothesis of CPSP and Its Clinical Implications

In light of the present data, we propose that the P2X7 hypothesis of CPSP explains the multiple mechanisms involved in modulating the pain responses of CPSP in terms of behavioral, molecular, and electrophysiological parameters. For example, P2X7 KO reduced thermal and mechanical pain, astrocytes, microglia, and BDNF expression. The data indicated that P2X7 receptor KO seemed to reduce CPSP-induced neuroinflammation. In the electrophysiological data, P2X7 receptor KO increased CSD amplitude compared to the CPSP group in the ACC; however, P2X7 receptor KO decreased unit numbers compared to the CPSP group in the fast component for the MD. Therefore, this neural pathway of the MD hypoactivity and ACC hyperactivity was due to ameliorating pain responses for CPSP symptoms. In the MEQ data, P2X7 KO attenuated the lower influx of chloride ions in the cytosol, reducing depolarization and CPSP symptoms.

In summary, the P2X7 hypothesis of CPSP suggests that reducing the lower chloride ion influx into the cytosol and its hyperpolarization results in the amelioration of CPSP symptoms. This hypothesis can be considered for the development of novel clinical treatments.

## 4. Materials and Methods

### 4.1. Animals

One hundred and six C57BL/6J mice were purchased from the National Laboratory for Animal Breeding and Research Center, Taipei, Taiwan. Fifty-one P2X7 KO mice (B6.129P2-P2rx7tm1Gab/J) were purchased from the Jackson Laboratory in the USA. All mice weighed 25–30 g at the beginning of the experiments. Mice were housed in groups of five in ventilated cages (21–23 °C, 50% humidity, 12-h light/dark cycles starting at 08:00 h). Food and water were provided ad libitum. All experiments were performed in accordance with the guidelines of the Academia Sinica Institutional Animal Care and Utilization Committee. Efforts were made to minimize both the number of animals used and the suffering of the animals.

### 4.2. Experimental Procedure

**Figure 8:**
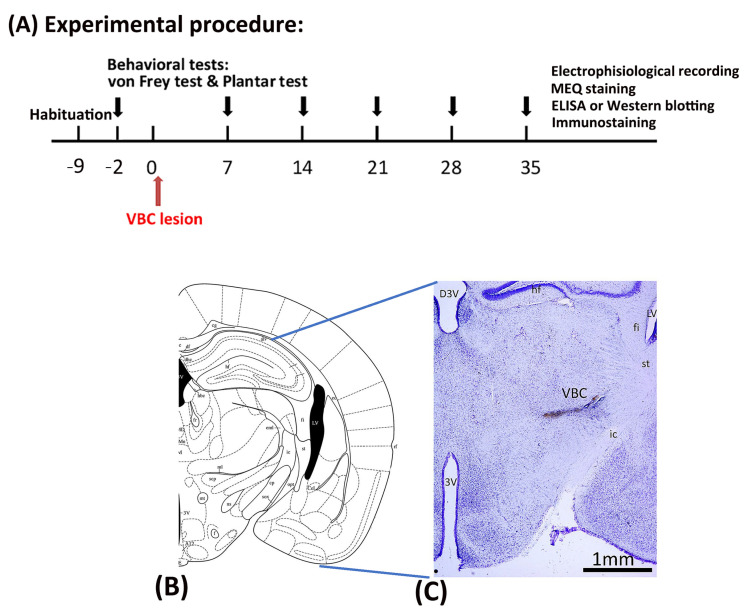
Mechanical pain and thermal pain were tested after thalamic hemorrhagic lesion induction. (**A**) The experiment was conducted. First, all mice were tested for mechanical pain via the von Frey test and thermal pain via the plantar test at baseline before inducing the VBC lesion of the thalamus. The VBC nuclei were destroyed on Day 0. The behavioral tests (i.e., the von Frey and plantar tests) were performed on Days 7, 14, 21, 28, and 35. After the behavioral tests, electrophysiological recordings, MEQ staining, ELISA, Western blotting, and immunostaining were performed. (**B**) Examples of collagenase lesions in the VBC. (**C**) Histological images of the VBC lesion. VBC: ventrobasal complex nuclei (i.e., the ventral posteromedial [VPM] and ventral posterolateral [VPL] portions).

All mice underwent a habituation procedure for 9 days at the beginning of the study. Seven days later, behavioral tests such as the von Frey and plantar tests were conducted, serving as the baseline. After 2 days, all mice received anesthesia and surgery to destroy the VBC of the thalamus. Mechanical pain was measured via the von Frey test and thermal pain via the plantar test on Days 0, 7, 14, 21, 28, and 35. On Day 35, immunostaining, electrophysiological recordings, MEQ recordings, ELISA, and Western blotting were performed after completing the behavioral tests (Figure 8A).

### 4.3. Surgery

After the 9-day habituation phase, all mice were given anesthesia and operated on to destroy the VBC (including the ventral posteromedial and ventral posteromedial nuclei of the thalamus), resulting in CPSP symptoms (Figure 8B,C). Twenty minutes prior to anesthesia, each mouse was intraperitoneally injected with atropine sulfate (0.1 mg) and gentamicin (6 mg). The mice were then anesthetized with sodium pentobarbital (50 mg/kg, i.p.). The C57BL/6J mice were assigned to the control and CPSP groups. The P2X7 KO mice were assigned to the P2X7/CPSP group. The VBC lesion group received 0.5 μL of 0.020 U collagenase type IV (Sigma, St. Louis, MO, USA), which was injected into the right VBC of the thalamus (anterior/posterior, −2.00 mm from the bregma; lateral, 1.8 mm from the midline; ventral, 3.00–3.20 mm from the skull surface). The control group received the vehicle Tris (hydroxymethyl)-methyl-2-aminoethane sulfonate (TES) buffer. The injection rate was 0.25 μL/min, and the needle was left in place for an additional 10 min. After surgery, the mice were allowed to recover for 7 days. During the recovery period, all mice were given food and water ad libitum.

### 4.4. Behavioral Tests

#### 4.4.1. Von Frey Test

Before testing, the mice were given habituation training in the von Frey task for 30 min. Von Frey filaments were gradually applied with top-down, graded force (2.0, 1.4, 1.0, 0.6, 0.4, 0.16, 0.07, 0.004, 0.02, and 0.008 g) to determine the minimal force that elicited a limb withdrawal response. The threshold was defined as the average of three minimal points measured in consecutive trials, with a 5-min inter-trial interval.

#### 4.4.2. Plantar Test

The mice were habituated to a transparent Plexiglas box for 30 min before testing. The plantar test was performed using radiant heating (IITC 390 G Plantar Test, IITC Life Science, Woodland Hills, Los Angeles, CA, USA). A focused heat light beam source stimulated the hind paw to elicit noxious withdrawal responses. The paw withdrawal response latency after heat stimulation was recorded. The right and left hind paws of each mouse were tested thrice, at 5-min intervals.

### 4.5. Recording of Evoked Multichannel Field Potentials and Unit Activities

Electrophysiological recordings were performed 5 weeks after hemorrhage lesion induction in the thalamus. Electrophysiological data were collected using a multichannel data acquisition system (Tucker-Davis Technologies, Alachua, FL, USA). The local field potential data recording sampling rate was 6 kHz, and the multiunit data recording sampling rate was 24 kHz. Extracellular field potentials and multiunit data were recorded with a multichannel probe (NeuroNexus, Ann Arbor, MI, USA) under maintenance anesthesia with 1–1.2% isoflurane-mixed oxygenated air. For local field potential and multiunit recordings, electrodes were placed in the right ACC (2 mm anterior and 0.5–1 mm lateral to the bregma) and in the right MD (2–2.5 mm posterior and 0.5–1.0 mm lateral to the bregma). The left sciatic nerve was isolated and implanted with a stainless-steel wire electrode to deliver constant-current pulses (pause duration = 0.5 ms, inter-pause duration = 10 s; Model 2100, A-M Systems, Carlsborg, WA, USA) for sciatic nerve stimulation (SNS). The minimal effective pulse current that could elicit hind limb vibration was measured and recognized as the 1-fold threshold stimulation strength. Local field potential activity in the ACC was recorded using 5-, 10-, and 20-fold increases in the threshold current strength. Neuronal multiunit activity in the MD was recorded using 1-, 2-, 5-, 10-, and 20-fold increases in the threshold current strength. The present study used CSD analysis to determine the underlying spontaneous current generator oscillations or evoked field potentials [37,38]. According to neuron morphology and coordination, mathematical analysis could reconstruct the ion current flow between cortical layers [17]. The membrane current, *Im*, was derived from the second spatial derivations of the extracellular field potentials and was calculated by the simplified formula

$$Im=\frac{\left[f\left(x-2h\right)-2f\left(x\right)+f\left(x+2h\right)\right]}{{4h}^{2}}$$

where *h* is the distance between successive measuring points (100 μm in the present study) and *x* is the coordinate perpendicular to the cortical layer. The present CSD trace was the grand average of 20 sweeps of spontaneous or evoked local field potential. Multiunit activities of the evoked responses were obtained by filtering the local field potentials (200–2000 Hz). Spikes that exceeded spontaneous background noise by 4-fold were marked as the digital signal “1” and subthreshold signals were marked as the digital signal “0”. Twenty multiunit activity trials were summated and presented in a post-stimulus histogram. To monitor neuronal activity over a long period, wave spikes over 4-fold of the background of the spontaneous local field potential were recorded with a 24 kHz sampling rate. The multiunit spike activities in the thalamus and the calculated numbers of spikes were analyzed in the present study.

### 4.6. Immunohistochemical Staining

After the behavioral tests and electrophysiological recordings, the animals were sacrificed and perfused with 4% paraformaldehyde in 0.1% phosphate-buffered saline. The mouse brain was collected and post-fixed at 4 °C. Brain tissue was incubated with a 30% sucrose/saline solution before cryo-sectioning (30 µm). The cryo-sections that contained ACC, MD, and VB were divided into three components. One component of the sections was stained with cresyl violet, and the other two components were stained with the following primary antibodies: mouse anti-IBA1 (1:300, MABN92, Millipore, South San Francisco, CA, USA), mouse anti-NeuN (1:400, MAB377, Millipore), and rabbit anti-GFAP (1:400, GTX108711, Genetex, Irvine, CA, USA). The secondary antibodies used were Alexa Fluor-488 goat anti-mouse IgG (H1L) antibody (1:1000, A11001, Life Technologies, Carlsbad, CA, USA) and Alexa Fluor-568 goat anti-rabbit IgG (H1L) antibody (1:1000, A11011, Life Technologies). Following immunohistochemistry, four sections with visible lesions from the center were chosen for image analysis. Stacks of images with 2-mm increments in depth were collected using a fluorescence microscope (BX51, Olympus, Waltham, MA, USA). The continual disruption of tissue organization and/or loss of staining were identified as the lesion area that should cover the lateral thalamic nucleus. The edges of the lesion were marked in individual sections, 100-µm distances from the edges of the lesion were chosen as the distant fields, and a 300 × 300 µm^2^ area was the region of interest measured.

### 4.7. Western Blot and ELISA

Tissue samples from the medial thalamus were used for these assessments. The tissues were lysed in lysis buffer (1% Triton X-100 with a complete protease inhibitor cocktail [Roche] in phosphate-buffered saline with pH 7.4). Lysates were clarified by centrifugation at 12,000 rpm for 15 min. The titration of protein concentrations was performed using the Pierce BCA protein assay kit method (Thermo, Waltham, MA, USA). The loading amount of each sample was 20 mg for Western blotting. Proteins were separated via 6–8% sodium dodecyl sulfate-polyacrylamide gel electrophoresis (SDS-PAGE; 6% SDS-PAGE for NKCC1 separation) and then transferred to a polyvinylidene difluoride (PVDF) membrane (Millipore). After blocking with 5% skim milk/TBST for 1 h at room temperature, the membranes were incubated overnight at 4 °C with the following primary antibodies diluted in 0.5% nonfat skim milk/TBST: rabbit anti-KCC2 (1:1000, Millipore), rabbit anti-NKCC1 (1:400, Abcam, Hong Kong, China), rabbit anti-TrkB (1:1000, Abcam), rabbit anti-GABAa a2 subunit (1:1000, Abcam), rabbit anti-P2X4 (1:400, APR-002, Alomone, Jerusalem, Israel), and mouse anti-tubulin (1:2500, Sigma). The membranes were washed three times with TBST and incubated with species-specific horseradish peroxidase-conjugated secondary antibodies for 45 min at room temperature. Then, the protein levels on the membranes were revealed using an electrochemiluminescence kit (Super Signal West Pico kit, Thermo). The expression of BDNF in TH peri-lesion tissue (i.e., the medial thalamus) was measured with an ELISA kit (ab212166, Abcam). The detection range of the standard curve was between 0 and 1000 pg/mL. For ELISA, the loading amount of each sample was 0.4 µg in 1 µL. The detected BDNF expression in whole samples was between 150 and 800 pg/mL.

### 4.8. Brain Slice Preparation and MEQ Induction

Fresh mouse brain was sliced into 250–300 μm slices and incubated in aCSF with 95% O_2_/5% CO_2_. After recovery, the slices were incubated with ~300 µM di-H-MEQ/ethyl acetate in aCSF for 30 min and then transferred to normal aCSF (Inglefield, 1999). Fluorescence images were captured using a fluorescence microscope (AxioImager Z1, Zeiss, Macquarie Park, NSW, Australia), Zen software (Zeiss ZEN 2.3; Zeiss), and a 20× water immersion objective (Achroplan 20×/0.5 W Ph2) for image capture. The excitation and emission wavelengths were 360 nm and 460 nm, respectively, capturing a series of images at 5-s intervals for about 30 min. To observe cytoplasmic chloride changes, 100 µM muscimol/aCSF was filled into the incubation chamber for 3 min and washed out with normal aCSF. The series image data were analyzed by ImageJ and the fluorescence intensity of each neuron of interest was measured individually. The relative power of the fluorescence intensity *F*(*S*) of neurons of interest in the % scale was defined as

$$F\left(S\right)i=\frac{Psi}{Ps1}\times 100$$

where *P*s1 = the intensity of the neuron of interest in the first image, *Psi* = the intensity of the neuron of interest in the image series *i*, and *i* = 1 to end.

Considering the variation in the degradation of each neuron, the original fluorescence intensity raw trace would convert to a 50–100% ratio distribution, with the formula being

$$F\left(CS\right)i=\frac{50}{F\left(S\right)1-F(S)end}\times \left(F\left(S\right)i-F\left(S\right)end\right)+50$$

where *F*(*CS*) = conversion of *F*(*S*) to a 50–100% ratio distribution, *F*(*S*)1, *F*(*S*)*i*, *F*(*S*)*end* = intensity of the neuron of interest in the first time series and the end of the image, and *i* = 1 to end.

### 4.9. Drugs

Type IV collagenase was obtained from Sigma-Aldrich (St. Louis, MO, USA). Muscimol was bought from Tocris (Bristol, UK). Collagenase was dissolved in saline, and the concentration was 40 U/mL. The dose of collagenase was 0.020 U, and the injection volume was 0.5 µL. A total of 100 µM muscimol was dissolved in normal saline.

### 4.10. Statistical Analysis

A mixed two-way ANOVA was conducted for the mechanical pain response in the von Frey test, thermal pain response in the plantar test, CSD amplitude and CSD peak latency in the ACC recording, fast component and late component in the MD recording, multiunit numbers per minute pre-/post-muscimol injections, and abnormal [Cl^−^] accumulation for F(CS)%. A one-way ANOVA was performed for the expression of NeuN, GFAP, IBA1, BDNF, TrkB, KCC2, NKCC1, GABAa, and P2X4. When appropriate, Tukey’s post hoc test was applied. Values of *p* < 0.05 were considered statistically significant.

## Data Availability

The original contributions presented in the study are included in the article, further inquiries can be directed to the corresponding authors.

## Abbreviations

ACC, anterior cingulate cortex; ATP, adenosine triphosphate; ANOVA, analysis of variance; BDNF, brain-derived neurotrophic factor; CPSP, central post-stroke pain; GFAP, glial fibrillary acidic protein; IBA1, ionized calcium-binding adaptor molecule 1; KO, knockout; MED1, mediator complex subunit 1; NeuN, neuronal nuclear antigen; TrkB, tropomyosin-related kinase receptor B; VBC, ventrobasal complex.
